# Blackcurrant Anthocyanins Attenuate Estrogen -Deficiency-Induced Bone Loss through Modulating Microbial-Derived Short-Chain Carboxylic Acids and Phytoestrogen Metabolites in Peri- and Early Postmenopausal Women

**DOI:** 10.3390/metabo14100541

**Published:** 2024-10-11

**Authors:** Briana M. Nosal, Staci N. Thornton, Alexey V. Melnik, Ali Lotfi, Manije Darooghegi Mofrad, Alexander Aksenov, Elaine Choung-Hee Lee, Ock K. Chun

**Affiliations:** 1 Department of Nutritional Sciences, University of Connecticut, Storrs, CT 06269, USA; briana.nosal@uconn.edu (B.M.N.); manije.darooghegi_mofrad@uconn.edu (M.D.M.); 2 Department of Kinesiology, University of Connecticut, Storrs, CT 06269, USA; staci.thornton@uconn.edu (S.N.T.); elaine.c.lee@uconn.edu (E.C.-H.L.); 3 Department of Chemistry, University of Connecticut, Storrs, CT 06269, USA; alexey.melnik@uconn.edu (A.V.M.); ali.lotfi@uconn.edu (A.L.); alexander.aksenov@uconn.edu (A.A.); 4 Arome Science Inc., Farmington, CT 06032, USA

**Keywords:** blackcurrant, anthocyanins, bone mineral density, osteoporosis, menopause, women, short-chain carboxylic acids, phytoestrogens

## Abstract

Objectives: The present study aimed to assess the effects of blackcurrant (BC) anthocyanins on concentrations of microbial-derived short-chain carboxylic acids (SCCAs) and metabolites of phytoestrogens. We then examined their associations with six-month changes in whole-body bone mineral density (BMD) and biomarkers of bone metabolism. Methods: Fecal and blood samples from a pilot randomized controlled trial were collected and analyzed from 37 eligible peri- and early postmenopausal women aged 45–60 years who were randomized into one of three treatment groups consuming one placebo capsule (control), 392 mg BC (low BC) or 784 mg BC (high BC) daily for six months. Results: Significant differences were observed between groups at baseline in acetic, propionic, valeric, caproic and heptanoic acids (*p* < 0.05). Isobutyric acid significantly decreased from baseline (0 months) to six months in the control group (*p* < 0.05) and the high BC group had a significantly greater concentration than the control group at six months (*p* < 0.05). Butyric acid was significantly greater in the high BC group than low BC at six months (*p* < 0.05). Six-month changes in caproic and isobutyric acids showed weak correlations with changes in whole-body BMD (r = 0.3519, *p* < 0.05 and r = 0.3465, *p* < 0.05, respectively). Isovaleric and valeric acids displayed weak correlations with BALP (r = 0.3361, *p* < 0.05) and OPG (r = 0.3593, *p* < 0.05), respectively. Enterodiol was positively correlated with BALP (r = 0.6056, *p* < 0.01) while enterolactone was positively correlated with osteocalcin (r = 0.5902, *p* < 0.001) and negatively correlated with sclerostin (r = −0.3485, *p* < 0.05). Conclusions: The results suggest that BC may be a potential dietary agent to reduce postmenopausal bone loss through modulating microbially-derived SCCAs and phytoestrogen metabolites.

## 1. Introduction

Postmenopausal osteoporosis (PMO) is a major health concern affecting approximately 27% of women 65 and older [1]. During the menopausal transition, decreased estrogen levels lead to bone resorption surpassing formation, resulting in bone loss. PMO is an estrogen-deficiency-induced skeletal disorder characterized by reduced bone strength, increasing the risk for fracture in postmenopausal women [2].

The FDA has approved various medications for the treatment of osteoporosis, including antiresorptive agent bisphosphonates, estrogen-related therapies, parathyroid hormone analogs, denosumab, a RANKL inhibitor, and romosozumab, a sclerostin inhibitor [3]. However, the prescription of these medications has declined over the past decade, and this may, in part, be related to fear of adverse effects, among other factors [4]. Due to this, there is an increased focus on dietary alternatives for preventing osteoporosis that are both safe and effective.

Blackcurrants (Ribes nigrum; BC), particularly, have drawn our attention because we previously found they contain the greatest amount of anthocyanins (ACNs) among commonly consumed berries [5]. ACNs, a class of polyphenolic compounds, are potent antioxidant and anti-inflammatory compounds and have been implicated to have therapeutic effects in chronic diseases, namely cardiovascular diseases, obesity, diabetes, neurodegenerative diseases, cancer and bone loss [6,7,8]. ACNs have demonstrated beneficial roles in bone formation through the upregulation of osteoblastic genes and promoting the proliferation of osteoblasts while also playing an essential role in the downregulation of osteoclastogenesis through various mechanisms, including reducing reactive oxygen species (ROS) production and influencing the nuclear factor-κB (NF-κB) pathway [9,10]. In addition to the positive effects on bone, the health-promoting effects of ACNs have been suggested to be associated with the modulation of the gut microbiome [11]. The gut microbiome additionally plays a vital role in the metabolism of ACNs and produces metabolites that can impact bone health [8].

Bone loss related to PMO has been shown to be related to host immunity, which the gut microbiome may influence, and thus, the gut may be a potential target for the prophylaxis and treatment of PMO [12]. Gut dysbiosis has been connected to osteoporosis, highlighting one component of the relationship between the gut microbiome and bone [13]. The regulation of the gut microbiota is thought to be shaped by host-related factors and environmental factors, including diet, and the microbiome regulates bone metabolism through the immune and endocrine systems as well as influencing calcium balance [12]. Modifications to the gut microbiota can influence bone biology and material properties in addition to being implicated in the pathology of osteoporosis [12,14,15].

The gut microbiota also produce beneficial metabolites, including short-chain carboxylic acids (SCCAs), which have been implicated as a key connector of the gut and bone [16]. SCCAs are a class of metabolites produced following the fermentation of indigestible carbohydrates or amino acids by microbiota [14], the most common of which are acetic acid, propionic acid and butyric acid, comprising approximately 90–95% of SCCAs in the human colon [17]. The fermentation of ACNs predominately contributes to the generation of lactic, acetic, propionic and butyric acids [18]. BC has also been shown to increase the formation of propionic and butyric acids in rats [19]. Overall, ACNs contribute to reshaping the microbial community and may primarily function as prebiotic agents due to their low bioavailability [20] while also influencing SCCA composition [11].

Phytoestrogens are secondary plant metabolites structurally and functionally similar to mammalian estrogens [21]. The gut microbiome converts plant-derived lignans to mammalian enterolignans, enterodiol and enterolactone [22]. Phytoestrogens have been shown to have various health benefits in humans; however, few studies have evaluated their effect on bone health [23,24,25]. Due to similarities with mammalian estrogen, it may be hypothesized that these compounds may counteract decreases in estrogen associated with the menopausal transition.

Our previous human findings indicated that BC supplementation for six months reduced the loss of whole-body BMD and significantly increased the concentration of serum amino-terminal propeptide of type 1 procollagen (P1NP), a biomarker of bone formation [26], while marginally significantly decreasing the receptor activator of nuclear factor kappa-Β ligand (RANKL, only in the high BC group) (*p* = 0.052) [27]. We also identified six bacteria correlated with BMD changes in the high BC group (*p* < 0.05), suggesting that BC’s bone protective effects might be driven via the gut–bone axis. Thus, the current study aimed first to evaluate the effects of BC ACNs on concentrations of microbially-derived SCCAs and phytoestrogen metabolites and then to assess their associations with six-month changes in whole-body BMD and biomarkers of bone metabolism.

## 2. Materials and Methods

### 2.1. Study Design

This six-month pilot randomized, double-blind, placebo-controlled, 3-arm clinical trial was conducted in peri- and early postmenopausal women to determine the effects of BC supplementation on bone health and the gut microbiome (Clinical Trials NCT04431960). A detailed description of the study design and bone data has been previously published [26]. Briefly, participants were recruited with advertisements in newspapers, flyers and emails from northeastern Connecticut. The inclusion criteria were as follows: (1) women aged 45–60 years, (2) not taking hormone replacement therapy for at least one year before beginning the study and (3) maintain normal exercise levels (<7 h/week). Exclusion criteria included the following: (1) major chronic disease(s), (2) heavy smokers, (3) any plan or chance of pregnancy, (4) taking prescriptions that may alter bone and calcium metabolism and/or anabolic agents or (5) heavy alcohol consumption (>2 drinks/day or a total of 12 drinks/week). Participants were randomized into one of three groups: group 1 consumed one placebo capsule per day (control), group 2 consumed one 392 mg BC capsule per day (low BC) and group 3 consumed two 392 mg BC capsules per day totaling 784 mg (high BC). Each 392 mg capsule of BC extract contained 176 mg of ACNs. The origin and full ACN content have been previously reported [26].

**Figure 1:**
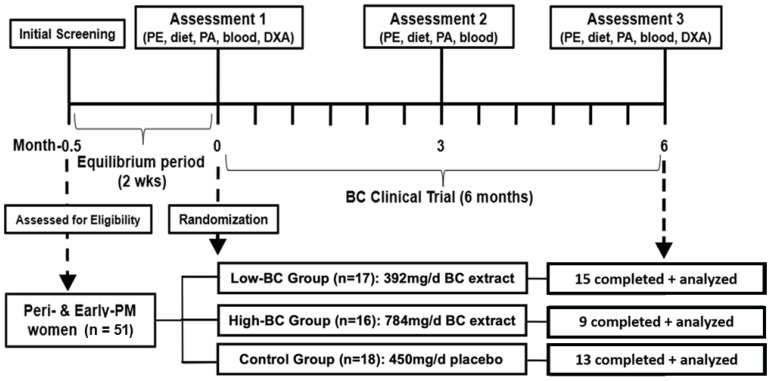
Flow diagram of study design.

All study procedures were performed at the Human Performance Laboratory in the Department of Kinesiology at the University of Connecticut Storrs Campus from July 2021 to October 2022. As shown in Figure 1, 51 women were eligible for participation in the study after giving their written consent. A baseline interview was conducted to collect medical history and diet behaviors followed by an initial examination of anthropometric measures and blood pressure. Following the initial visit, subjects began a two-week equilibrium period where they began taking a supplement containing 400 mg calcium and 500 IU vitamin D (Bayer AG, Leverkusen, Germany) to avoid any potential bone deterioration. Following equilibration, participants were randomly assigned to one of the three treatment groups for 6 months.

Participants visited the study center at three additional time points following their initial visit at 0 months (baseline), 3 months and 6 months. At month 0, participants completed additional consent forms to confirm their eligibility and at months 0 and 6 were required to have a negative urine pregnancy test before completing the dual x-ray absorptiometry (DXA) scan. At months 0, 3 and 6, a physical examination was conducted to assess anthropometrics and blood pressure, 12 h fasting blood and fecal samples, and a 3-day food record and physical activity log were collected based on the week prior to each study visit. For the duration of the study, participants were asked to refrain from taking any dietary supplements other than those provided and to maintain normal eating habits apart from avoiding ACN-rich foods and fermented dairy products that contained bifidobacteria or lactobacilli.

The final number of participants included in the current analysis was 37. Of the initial 51, 11 dropped due to personal reasons unrelated to the intervention drugs; specific reasons for dropouts are listed elsewhere [26]. An additional 3 participants were excluded from the current analysis due to lack of samples available to complete metabolomic analyses.

Compliance throughout the duration of the study was monitored via interview and by counting the unused portions of intervention drugs at each study visit. Compliance was defined as missing ≤2 doses per week [28] and the overall compliance for all three groups was ≥95% [26]. Prior to the initiation of the project, the proposed project and procedures were reviewed and approved by the University of Connecticut Institutional Review Board (#HR20-0035).

### 2.2. Biospecimen Analysis

#### 2.2.1. Fecal Sample Fixation and Storage

Fecal samples were collected by participants using DNA Genotek OMR-200.100 kits (DNA Genotek, Stittsville, ON, Canada) designed for stool collection and preservation. Upon arrival at the study center, stool kits were collected, and samples were frozen at −80 °C and stored under these conditions until processed and analyzed. Rapid processing on ice was performed to minimize degradation of volatile compounds.

#### 2.2.2. Quantification of Microbial-Derived Short-Chain Carboxylic Acids

SCCA analysis was performed using fecal samples collected at months 0 and 6. First, the fecal samples were weighed (50–100 mg) then cold 100% methanol was added (10 µL/mg of sample). The samples were then vortexed for 10 min, sonicated for 10 min and centrifuged at 14,000 rpm for 10 min at room temperature. An amount of 100 µL of supernatant was then transferred to vials for gas chromatography mass spectrometry (GC-MS) analysis. GC-MS analysis was carried out using an Agilent GC-MSD with an HP-FFAP column and a temperature gradient as follows: starting at 50 °C and increasing by 15 °C per minute until reaching 220 °C, the MS signal was acquired in scan mode over a 50–500 Da *m*/*z* window. The targeted peaks for the SCCAs were extracted using Agilent quantification software and external calibration for eight SCCAs (acetic acid, propionic acid, isobutyric acid, butyric acid, isovaleric acid, valeric acid, caproic acid and heptanoic acid) were used to quantify the peaks in samples. This targeted analysis was performed using selected ion monitoring for individual fragment masses of each compound. We screened only the targeted *m*/*z* values and fragment patterns of interest.

#### 2.2.3. Quantification of Phytoestrogen Metabolites

Phytoestrogen metabolites, enterodiol and enterolactone, were measured using targeted metabolomics analysis in fecal samples collected at months 0 and 6. First, fecal samples were weighed (50–100 mg) and chilled 60% methanol was added to each sample (10 µL/mg of sample). The samples were then sonicated on ice for 10 min followed by vortexing for 2 h at 4 °C. Following vortexing, the samples were sonicated on ice for 10 min then centrifuged at 14,000 rpm for 5 min at room temperature. An amount of 300 µL of supernatant was transferred to a new vial and 600 µL of an extraction buffer (50 µM) containing cold 100% methanol with internal standards, sulfadimethoxine (5 mM) and sulfachloropyradazine (SCP) (5 mM). Following the addition of the extraction buffer, samples were vortexed for 30 s, sonicated on ice for 5 min and centrifuged at 12,000 rpm for 10 min at room temperature. An amount of 300 µL of this sample was then lyophilized for approximately 2 h and resuspended using 100 µL of a resuspension buffer (50 µM) containing 10% acetonitrile and internal standards, sulfamethizole (5 mM) and sulfamethazine (5 mM). The samples were then sonicated on ice for 5 min, vortexed for 30 s and the supernatant was transferred for injection.

Targeted metabolites were then measured and analyzed using ultra-high performance liquid chromatography/mass spectrometry (uHPLC-MS) for injecting samples and chromatographically separating using a C18 chromatography column (50 mm × 2.1 mm Kinetex1.7 μM, C18, 100 Å), 40 °C column temperature, 0.4 mL/min flow rate, mobile phase A 99.9% water (Fisher Scientific, Waltham, MA, USA), 0.1% formic acid (Thermo Fisher Scientific, Waltham, MA, USA), mobile phase B 99.9% acetonitrile (Fisher Scientific, Waltham, MA, USA) and 0.1% formic acid (Fisher Scientific, Waltham, MA, USA). MS analysis in negative and positive polarity mode was performed on an Agilent 6545 QTOF mass spectrometer equipped with Dual Jet ESI sources (Agilent Technologies, Santa Clara, CA, USA).

#### 2.2.4. Quantification of Biomarkers of Bone Metabolism

Twelve-hour fasting blood samples were collected and analyzed from months 0 and 6. The blood (80 mL) was used to determine changes in biomarkers of bone metabolism. Plasma samples were collected in heparin tubes and serum in SST tubes (BD Vacutainer, Mississauga, ON, Canada). Whole blood samples were centrifuged at 3000 g for 15 min at 4 °C, and 500 µL of serum and plasma were subsequently aliquoted and stored at −80 °C until analyzed. Changes in BALP, osteocalcin and sclerostin were measured in heparin plasma and OPG, RANKL and IGF-1 were measured in serum at months 0 and 6 using commercially available ELISA kits.

### 2.3. Bone Density Measurements

Whole-body BMD was measured via DXA scans at months 0 and 6.

### 2.4. Statistical Analysis

Fecal SCCAs were normalized using sample-specific normalization with sample weight on MetaboAnalyst 6.0 (https://www.metaboanalyst.ca, accessed on 2 February 2024). Phytoestrogen metabolites were quantified and normalized using Agilent MassHunter software (Version 10.0, Agilent Technologies, Santa Clara, CA, USA). The significance of differences in SCCA concentration at baseline, six months and baseline versus six months was assessed by repeated measures two-way ANOVA followed by Tukey’s post hoc test. The significance of differences in six-month percent changes in SCCAs and phytoestrogen metabolites was evaluated using one-way ANOVA. The correlations between six-month changes in SCCAs, phytoestrogen metabolites, BMD and biomarkers of bone metabolism were tested using Pearson’s correlation. All data were analyzed using SAS (Version 9.4, SAS Institute, Cary, NC, USA), and *p* < 0.05 was considered statistically significant.

## 3. Results

### 3.1. Baseline Characteristics

The baseline characteristics of the study population have been outlined in our previous study [26]. Briefly, there were no significant differences found between groups at baseline in age, anthropometric measures, blood pressure, physical activity, BMD measurements, biomarkers of bone metabolism or nutrient intake.

### 3.2. Analysis of Short-Chain Carboxylic Acids

#### 3.2.1. Concentrations at Baseline and Six Months

**Figure 2:**
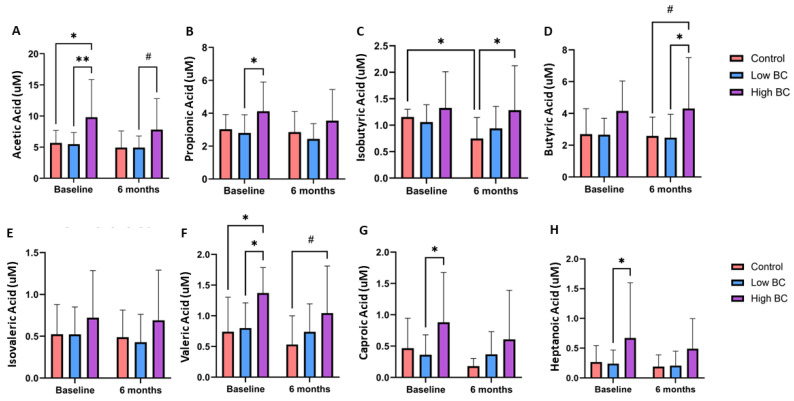
Baseline and six-month comparison of SCCA concentrations measured by GC-MS across three groups for (**A**) acetic acid, (**B**) propionic acid, (**C**) isobutyric acid, (**D**) butyric acid, (**E**) isovaleric acid, (**F**) valeric acid, (**G**) caproic acid and (**H**) heptanoic acid. Data are presented as mean ± SD. *n* = 13 control group, *n* = 15 low BC group and *n* = 9 high BC group. * *p* < 0.05, ** *p* < 0.01, # *p* = 0.05–0.09.

Figure 2 displays concentrations of fecal SCCAs measured by gas chromatography mass spectrometry (GC-MS) at baseline and six months. At baseline, before BC treatment, significant differences were observed between the high BC group and low BC group for acetic acid (*p* < 0.01), propionic acid (*p* < 0.05), valeric acid (*p* < 0.05), caproic acid (*p* < 0.05) and heptanoic acid (*p* < 0.05). Significant differences were observed between the high BC group and control group at baseline for acetic acid and valeric acid (*p* < 0.05). Isobutyric acid was significantly decreased from baseline to six months in the control group (*p* < 0.05) and the concentration in the high BC group was significantly greater than that of the control group at six months (*p* < 0.05). The concentration of butyric acid in the high BC group was significantly greater than that in the low BC group (*p* < 0.05) and marginally significantly greater than that in the control group (*p* = 0.0616) at six months. Acetic acid concentrations in the high BC group were marginally significantly greater than those in the low BC group (*p* = 0.0888), and valeric acid concentrations were marginally significantly greater than those in the control group (*p* = 0.0610) at six months.

#### 3.2.2. Correlations between Six-Month Changes in SCCAs, Whole-Body BMD and Biomarkers of Bone Metabolism

**Figure 3:**
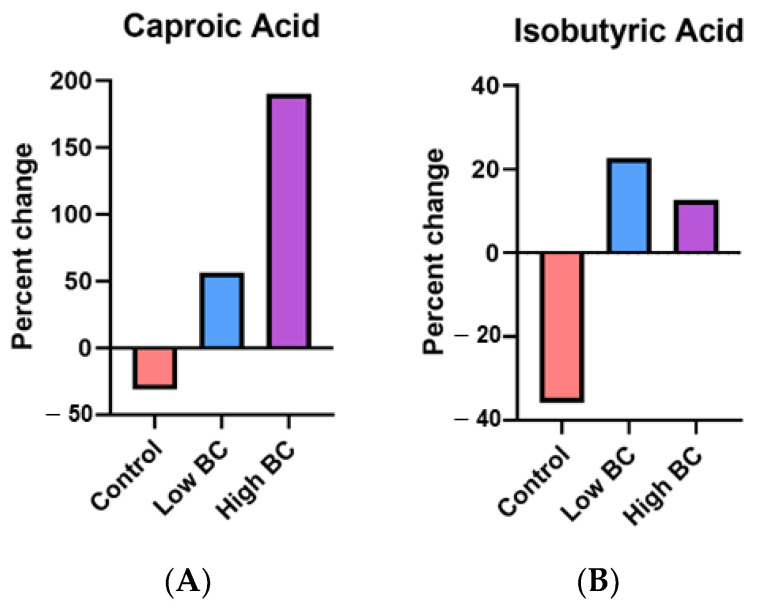
Six-month percent changes in caproic acid (**A**) and isobutyric acid (**B**).

Six-month percent changes in caproic acid showed an increasing trend across all groups with the high BC group having the greatest increase (Figure 3). Six-month percent changes in isobutyric acid also showed an increasing trend with both the low and high BC groups increasing while the control group displayed a decrease (Figure 3).

Table 1 displays the correlations between six-month changes in SCCAs with six-month changes in whole-body BMD and biomarkers of bone metabolism. Changes in caproic (r = 0.35192, *p* < 0.05) and isobutyric (r = 0.34648, *p* < 0.05) acids displayed weak correlations with whole-body BMD percent changes from baseline. Weak positive correlations were also observed between isovaleric acid and bone-specific alkaline phosphatase (BALP) (r = 0.33614, *p* < 0.05) and valeric acid with osteoprotegerin (OPG) (r = 0.35926, *p* < 0.05).

### 3.3. Analysis of Phytoestrogen Metabolites

#### 3.3.1. Six-Month Percent Changes

**Figure 4:**
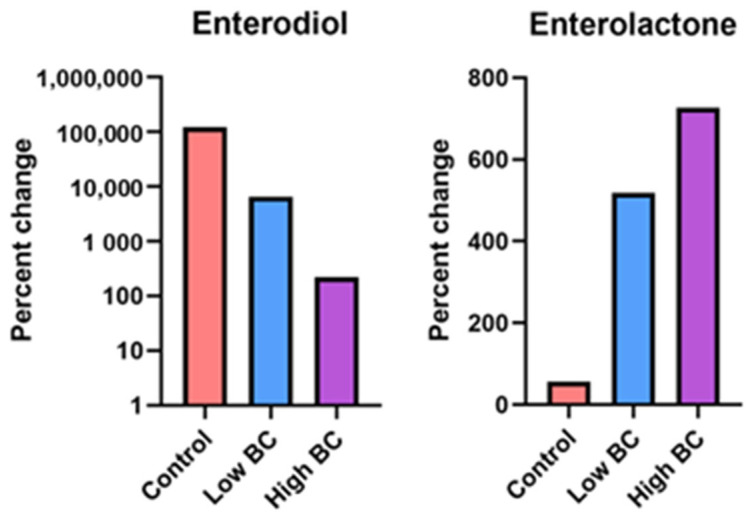
Six-month percent changes in phytoestrogen metabolites concentrations.

Although not significant, lignan-derived phytoestrogen metabolites, enterodiol and enterolactone, displayed opposite trends respective to six-month percent changes. Enterodiol displayed a decreasing tendency across groups while enterolactone displayed an increasing tendency (Figure 4).

#### 3.3.2. Correlations between Six-Month Changes in Phytoestrogen Metabolites and Biomarkers of Bone Metabolism

Table 2 displays the correlations between phytoestrogen metabolites and biomarkers of bone metabolism. Enterodiol was positively correlated with BALP (r = 0.60561, *p* < 0.01). Enterolactone was positively correlated with osteocalcin (r = 0.59022, *p* < 0.0001), and a weak negative correlation was observed with sclerostin (r = −0.34853, *p* < 0.05).

## 4. Discussion

Previous literature has shown that BC may effectively reduce bone loss in both in vitro [10,29,30,31,32] and in vivo experiments [29,32]. In our human studies, we also found that BC attenuated bone loss as shown by increased whole-body BMD and increased P1NP by BC treatment [26]. In the current study, we found that BC may exert these bone-protective effects through the modulation of SCCAs and phytoestrogen metabolites. However, these preliminary findings need to be further confirmed. SCCAs are a major class of metabolites produced by the gut due to the fermentation of dietary fibers following the consumption of whole grains, fruits and vegetables. BC bioactive components include ACNs, the most prominent parent ACNs being cyanidin [30,33] and delphinidin [33]. The fermentation of ACNs contributes to producing SCCAs, predominately lactic, acetic, propionic and butyric acids [18]. Overall, SCCAs have been implicated as a key link between the gut and bone [16].

In our six-month intervention, we found that butyric acid concentrations were significantly higher in the high BC group than in the low BC group and marginally higher than in the control group after six months of intervention. Additionally, acetic acid had a marginally significantly higher concentration in the high BC group compared to the low BC group at six months. A recent meta-analysis on the consumption of ACNs and their influence on gut health markers evaluated the results of animal studies assessing the effects of ACN-rich diets, largely using berries, on acetic, butyric and propionic acids using cecal matter [34]. In agreement with our findings, the authors found that in interventions lasting longer than 4 weeks, considerable increases were found in acetic acid concentrations. This study indicated that longer durations and higher dosages of ACNs were more effective in enhancing the levels of the SCCAs evaluated. Butyric, acetic and propionic acids are among the most widely studied SCCAs for bone health and have been shown to inhibit bone resorption in mice. An in vivo study using a postmenopausal bone loss model found both butyric and acetic acids can modulate bone metabolism through the suppression of osteoclast differentiation, thus reducing bone resorption [35]. They further found that butyrate significantly suppressed *TRAF6* and *NFATc1*, two key osteoclastogenic signaling components, after receptor activator of nuclear factor-κB ligand (RANKL) stimulation [35]. This suggests the increase in butyric acid in the current study may have contributed to the bone-protective effects of BC by reducing osteoclastogenic signaling.

The remaining SCCAs evaluated in the current study are less studied in the context of bone health. We found that caproic acid displayed a weak positive correlation with whole-body BMD changes over six months and displayed an increasing trend across groups relative to percent changes. A previous study found that caproic acid was among the SCCAs not correlated with BMD at any skeletal sites [36]. However, the authors evaluated BMD at the lumbar spine, total left hip and ultra-distal radius and ulna at a single time point, and the SCCAs were measured in serum samples while the current study evaluates whole-body BMD and fecal SCCAs. The role of valeric acid is not yet fully understood in the context of gut health [37]; however, we found valeric acid to have a marginally increased concentration in the high BC group compared to that in the control group at 6 months. Previously, valeric acid has been linked to the inhibition of osteoclast-like cell differentiation and the increased differentiation of pre-osteoblasts into osteoblasts with increased alkaline phosphatase activity (ALP) [36]. Valeric acid also decreased RELA protein production [36], which is a subunit of RANK and plays a crucial role in the pathogenesis of many inflammatory diseases [38]. Although we did not find any associations between valeric acid and RANKL or other inflammatory cytokines in the current study, we found that OPG and valeric acid showed a weak positive correlation. OPG is a protein secreted during bone turnover for its role as a decoy receptor for RANKL in osteoclasts [39] and acts as a soluble decoy receptor binding to RANKL in competition with RANK, which can inhibit osteoclastogenesis and is thus known as a osteoclasts inhibiting factor [40]. However, in inflammatory conditions, including estrogen deficiency, B cell dysregulation results in increased B cell RANKL expression, reduced OPG expression and a shift towards excessive osteoclastogenesis and thus, bone resorption [41]. The current finding suggests a potential relationship between these two compounds. However, the associations were relatively weak and should be interpreted cautiously. Additionally, these preliminary findings need to be further studied to confirm relationships.

Isobutyric and isovaleric acids are branched SCCAs that are produced by the fermentation of branched amino acids generated from undigested proteins reaching the colon [42]. Due to scarce research in relation to branched SCCAs and bone health, we may presume that isobutyric acid acts to influence bone health through influencing BMD; however, while the correlation observed between isobutyric acid and BMD was statistically significant, the associations were relatively weak, and further research is needed to explore this potential relationship and mechanisms. Isovaleric acid was weakly correlated with BALP, a biomarker of bone formation that is secreted by osteoblasts and plays a role in the propagation of bone mineralization, also accounting for up to half of the total blood ALP activity in adults [43]. Estrogen decline following ovariectomies in rats stimulates osteoclastic bone resorption and degradation of the bone matrix, resulting in increased BALP secretions in the blood [44]. Given that the current population is comprised of peri- and early postmenopausal women, we would expect that estrogen levels are beginning to decline, and this may be responsible for the increased blood BALP levels. Although the full mechanism of isovaleric acid influence on bone has not been fully elucidated, it has been shown to have a strong inhibitory effect on bone resorption in an ovariectomy-induced osteoporotic model through repressing osteoclast differentiation [45]. Some authors also found that in the mouse model, isovaleric acid upregulated the expression of genes associated with bone formation including *Osx* and *Runx2* [45]. In combination with our results, these findings suggest that in addition to the inhibitory effects on bone resorption, isovaleric acid may also influence BALP concentrations and the regulation of bone formation; however, further research is needed to confirm the current preliminary findings and elucidate potential mechanisms.

The bioavailability of dietary lignans is limited as they are not often absorbed in the small intestine or are excreted in urine [46,47]; however, a large fraction reaches the colon where they are metabolized to form mammalian lignans, enterodiol and enterolactone, through hydrolysis, dihydroxylation, demethylation and oxidation [48]. In the current study, we found that enterodiol percent changes displayed a decreasing trend across groups, with the control group having the greatest increase and the high BC group having the lowest. Opposingly, enterolactone showed an increasing trend across groups but at a lower magnitude than enterodiol. Few studies have evaluated the relationship between lignans and enterolignans with bone health. One estimated dietary intake of lignans and found enterolignans were positively associated with BMD in postmenopausal women but were nonsignificant when dietary calcium was included in the model [25]. Similarly, Kuhnle et al. found that enterolignan precursors were positively associated with BMD, but this was not maintained when calcium was considered in the model [24]. However, these studies evaluated dietary estimates of enterolignans and not levels from blood or fecal samples. In the current study, calcium and vitamin D supplements were provided to participants for the study, potentially leading to a nonsignificant association between enterodiol and enterolactone concentrations with BMD.

The strong opposing trends between enterodiol and enterolactone regarding six-month percent changes can be explained by the biochemical steps required to transform plant lignans into enterolignans. The plant lignan secoisolarisiresinol diglucoside (SDG) can be metabolized to enterodiol and enterolactone via secoislariciresinol; however, no single bacterium has been identified to metabolize SDG to enterolactone completely [49]. Different bacterial strains may also be involved in converting SDG to produce enterodiol, and further, enterolactone [50]. Jin et al. found that the production of enterolactone from enterodiol has been shown to be related to enantioselective oxidation by intestinal bacteria [51]. Both enterodiol and enterolactone have two enantiomeric forms, including a (−)-form and a (+)-form. The authors isolated a single bacterium, END-1, containing (−)-enterodiol, which was found to transform into (−)-enterolactone. Due to the lack of transformation of (+)-enterodiol with END-1, the strain END-2 was isolated and found to transform (+)-enterodiol to (+)-enterolactone. The authors determined that both END-1 and END-2 were *Ruminococcus* species. We previously found that BC dose-dependently increased the relative abundance of *Ruminococcous 2* which was one of six bacteria correlated with whole-body BMD changes in the high BC group [27]. We may presume that *Ruminococcus* was involved in the production of enterodiol and enterolactone in the current study. Several other bacterial groups, including *Clostridia*, likely play a role in the transformation of plant lignans to enterolignans, and thus, future research is needed to identify the bacterial strains related to the production of enterodiol and enterolactone in the current study population and the influence of BC supplementation on these bacteria. Furthermore, the determination of multiple bacterial strains involved in the transformation of enterodiol to enterolactone suggests that the metabolism of lignans is highly dependent on specific bacteria that exert enantioselective dihydroxylation and oxidation properties. Although anthocyanins and lignans are two distinct plant compounds, we may postulate that BC influence on the bacterial species present, inferred through targeted metabolomics, may have influenced the production of enterodiol and enterolactone.

We also found that enterodiol was positively correlated with BALP and enterolactone was positively correlated with osteocalcin. Previous literature has provided some evidence to suggest both enterodiol and enterolactone may influence ALP and osteocalcin. Feng et al. evaluated the effects of enterodiol and enterolactone on a human osteoblast-like cell line (MG-63 cells) and found that at lower concentrations, both enterolignans significantly increased MG-63 cell viability and ALP activity, while at higher concentrations, they increased the mRNA expression of osteocalcin [23]. The authors suggested that enterolactone and enterodiol may have biphasic effects on osteoblast-like cells, where at low doses, cell proliferation and ALP activity are induced, while inhibitory effects are seen at higher doses. A similar conclusion was drawn by Wang and Kurzer who reported that in breast cancer cells, enterolignans display biphasic effects [52]. Osteocalcin is an abundant protein in bone expressed in osteoblasts and has been widely used as a biomarker of bone turnover, indicative of a high remodeling status during PMO. However, Martiniakova et al. reported the results of meta-analyses comparing several markers of bone turnover, finding no significant differences in osteocalcin levels between healthy and osteoporotic individuals, suggesting osteocalcin may not be a good indicator of bone turnover in PMO [53]. We additionally observed a weak negative correlation between enterolactone and sclerostin. To our knowledge, there are no studies that have evaluated the relationship between enterolignans and sclerostin. Sclerostin is secreted mainly by osteocytes [54] but has been found in chondrocytes [54] and osteoclasts [55]. The involvement of sclerostin in the pathogenesis of many skeletal disorders has been outlined in various studies, and more recently, antisclerotic antibodies have been approved for the treatment of osteoporosis [56]. Although there is no clear evidence to suggest that osteocalcin is a good indicator of bone turnover and the mechanisms through which enterolactone may influence sclerostin concentration, enterodiol and enterolactone have pleiotropic effects on mechanisms involved in bone metabolism, and the results regarding BC’s influence on these enterolignans are promising but require further research.

The current study has several strengths. First, this study targeted peri- and early postmenopausal women, which is a critical period in menopausal transition and the most optimal to prevent bone loss before it has significantly progressed. This is also the only study, to our knowledge, that has evaluated the effects of BC supplementation on SCCAs and phytoestrogen concentrations in women. We identified SCCAs as well as enterolignans whose production may be influenced by the consumption of BC anthocyanins. However, some limitations do exist. First, the current study size was small leading to large variations in some of the data, and there was a lack in diversity with the majority of the study population being Caucasian, limiting the generalizability of the findings. The SCCA baseline data particularly displayed large variations. However, generally, SCCAs naturally exhibit high variability due to individual differences in diet and gut microbiome composition. Second, the participants were given a calcium and vitamin D supplement, which may have influenced some of the results. As previously mentioned, when calcium was included in the model when evaluating associations between BMD and enterolignans, the associations were no longer significant [23,24]. Lastly, the current study did not include blood measurements of SCCAs to determine differences between excretion versus absorption, and microbiome data were not used to confirm the bacterial species involved in the production of SCCAs and phytoestrogen metabolites.

## 5. Conclusions

The findings of this pilot study suggest that BC anthocyanins may influence changes in microbial-derived SCCA concentrations as shown by greater concentrations at 6 months observed in acetic acid, isobutyric acid, butyric acid and valeric acid in the high BC group compared to those in the control and low BC groups. Weak positive correlations were found between caproic and isobutyric acids with whole-body BMD and isovaleric and valeric acids with biomarkers of bone metabolism, suggesting that BC consumption may influence the mechanisms by which SCCAs can affect bone health, creating an avenue for further research. A decreasing trend in the percent change of enterodiol and an increase in enterolactone reflect the relationship between these two metabolites, with enterolactone being a secondary metabolite of enterodiol. Positive correlations between the bone formation markers BALP and osteocalcin with enterodiol and enterolactone, respectively, suggest the potential of these phytoestrogen metabolites to influence bone metabolism. Although the results of this pilot study include a small sample size and large variation in some findings, preventing clear conclusions, and although the associations were relatively weak and should be interpreted cautiously, the current study demonstrated that BC may be a potential dietary agent to attenuate postmenopausal bone loss through modulating microbially derived metabolites. Future studies are needed to evaluate the absorbance of SCCAs and phytoestrogen metabolites as well as microbiome data to evaluate the bacterial species that are responsible for the production of these SCCAs [37] and phytoestrogen metabolites, specifically enterolignans [51,57,58,59].

## Figures and Tables

**Table 1:** Correlations between six-month changes in SCCAs and biomarkers of bone metabolism.

	Δ BMD		Δ BALP		Δ OPG		Δ RANKL ^a^		Δ IGF-1 ^b^	
	r	*p*-Value	r	*p*-Value	r	*p*-Value	r	*p*-Value	r	*p*-Value
Δ ACE	0.00485	0.9779	0.10344	0.5483	0.08597	0.6181	0.25753	0.1272	−0.10278	0.5822
Δ PRO	−0.01731	0.9190	−0.06701	0.6978	0.19478	0.2550	−0.00083	0.9964	0.13690	0.4627
Δ ISOB	0.34648	<0.05	0.02982	0.8629	−0.04374	0.8001	0.04702	0.7983	−0.14890	0.4240
Δ BUT	0.03265	0.8501	0.00194	0.9910	0.06151	0.7215	−0.20003	0.2723	−0.01497	0.8226
Δ ISOV	−0.19110	0.2572	0.33614	<0.05	−0.00001	1.0000	0.18877	0.3008	−0.21444	0.2467
Δ VAL	−0.18396	0.2758	−0.14906	0.3856	0.35926	<0.05	−0.08251	0.6535	0.35251	0.0518
Δ CAP	0.35192	<0.05	−0.07268	0.6736	−0.05121	0.7667	−0.08251	0.6570	−0.10290	0.5817
Δ HEP	0.22639	0.1910	−0.11392	0.5083	−0.09711	0.5731	0.06225	0.7350	−0.11711	0.5304

BMD, whole-body bone mineral density; BALP, bone-specific alkaline phosphatase; OPG, osteoprotegerin; RANKL, receptor activator of NF-κB; IGF-1, insulin-like growth factor-1. ^a^ *n* = 32 for RANKL due to missing samples. ^b^ *n* = 31 for IGF-1 due to missing samples.

**Table 2:** Correlations between six-month changes in phytoestrogen metabolites and biomarkers of bone metabolism.

	Δ BALP		Δ Osteocalcin		Δ Sclerostin		Δ RANKL ^c^	
	r	*p*-Value	r	*p*-Value	r	*p*-Value	r	*p*-Value
Δ Enterodiol ^a^	0.60561	<0.01	−0.01224	0.9527	−0.03034	0.8830	0.02930	0.8970
Δ Enterolactone ^b^	−0.06664	0.6994	0.59022	<0.0001	−0.34854	<0.05	−0.03349	0.8556

BALP, bone-specific alkaline phosphatase; RANKL, receptor activator of NF-κB. ^a^ *n* = 32 for enterodiol due to missing samples. ^b^ *n* = 36 for enterolactone due to missing samples. ^c^ *n* = 32 for enterolactone due to missing samples.

## Data Availability

The raw data supporting the conclusions of this article will be made available by the authors on request.
